# Decision Making Processes and Outcomes

**DOI:** 10.1155/2013/367208

**Published:** 2013-10-24

**Authors:** Julie Hicks Patrick, Jenessa C. Steele, S. Melinda Spencer

**Affiliations:** ^1^Department of Psychology, West Virginia University, P.O. Box 6040, Morgantown, WV 26506-6040, USA; ^2^Department of Psychology, West Virginia University, Morgantown, WV 26506-6040, USA; ^3^Department of Psychology, Radford University, P.O. Box 6946, Radford, VA 24141, USA; ^4^Department of Health Promotion Education and Behavior, University of South Carolina, Columbia, SC 2920, USA

## Abstract

The primary aim of this study was to examine the contributions of individual characteristics and strategic processing to the prediction of decision quality. Data were provided by 176 adults, ages 18 to 93 years, who completed computerized decision-making vignettes and a battery of demographic and cognitive measures. We examined the relations among age, domain-specific experience, working memory, and three measures of strategic information search to the prediction of solution quality using a 4-step hierarchical linear regression analysis. Working memory and two measures of strategic processing uniquely contributed to the variance explained. Results are discussed in terms of potential advances to both theory and intervention efforts.

## 1. Introduction

### 1.1. Decision Making Processes and Outcomes

A significant body of research has examined problem solving and decision making performance in adulthood (see [1, 2] for reviews). Both problem solving and decision making are concerned with the ways in which people interpret problems, form goals, search information, and combine information to arrive at solutions. Researchers often employ think-aloud and other process-tracing techniques to investigate the processes governing information search and cessation [3, 4]. The extant literature demonstrates that relative to younger and middle-aged adults, older adults approach decision making with different goals, apply different heuristics, seek different amounts and types of information in the predecision phase, and offer different decisions (e.g., [4–6]). 

Research has examined several possible mechanisms to explain this age difference, including the role of cognitive resources (e.g., [5, 7]), the social context and personal experience [8, 9], affective context [10], and the decision domain [11]. Sophisticated studies have examined these factors individually and in combination [12]. For many decision tasks, basic and intermediate cognitive skills such as working memory and speed of processing often are the strongest predictor of decision outcome [13].

 Process-tracing techniques may allow a more thorough examination of task performance and strategic processing [3, 4, 14, 15]. In the standard decision making task, materials are structured to reflect those available in the real-world, similar to the ecologically-rich social vignettes used in the everyday problem solving approach. Although in actual real-world information searches, people are able to view all of the available information simultaneously, an advantage to the process-tracing technique is that one is able to directly examine how an adult: (a) selects important features, (b) combines the information obtained, (c) evaluates specific alternatives, and (d) arrives at an adequate decision [14–16]. Specifically, multiple measures are used to index task performance, including the amount of information searched and the order in which the information is searched. Indicators of the amount of information searched include time on task and the number of information cells viewed (i.e., thoroughness). Research shows that older adults often use less information than younger adults [4, 17]. 

 Strategic search is also examined in the decision making task. The two main types of search strategies have been termed compensatory and noncompensatory [4, 18]. Compensatory search is an exhaustive strategy that relies on mathematical averaging of the features for each alternative. Thus, compensatory searches require significant effort as most or all of the available information is read, processed, evaluated, and combined. Noncompensatory strategies reduce the number of viable alternatives quickly when the information load exceeds a person's ability to attend to the information volume. Noncompensatory strategies accomplish this reduction by focusing exclusively on a few alternatives which have high or acceptable values on a set of key attributes. Thus, noncompensatory strategies are more resource-efficient than compensatory search strategies [14, 18].

 One area that is often overlooked in decision making research concerns the value of the information participants choose to view. However, information quality has been studied in examinations of expert decisions [19]. For example, some studies of experts have shown that they search very little of the available information before making a decision, in some cases viewing only one or two pieces of highly relevant information [15]. The experts are able to “zero in” on the most critical pieces of information to make a high quality decision. If the value of any given piece of information is known, researchers can measure the selectivity with which decision makers choose information. Using search selectivity, the quality of information used can be separated from the amount used and from the order of search. Thus, selectivity allows one to interpret differences in thoroughness and strategy.

### 1.2. The Current Study

 The current study extends investigations of strategic information search by focusing on both information quality and decision quality among a large sample of community-dwelling adults. By combining a focus on the contributions of individual characteristics of experience and working memory from the everyday problem solving literature with a focus on direct task performance measures from the decision making domain, the current study contributes to the literature by clarifying the roles of these predictors. By examining the ways in which differences in the information search process relate to differences in decision outcome, we can better understand age-related differences in decision making. Based on the everyday problem-solving literature, we expected to find age differences in decision quality, with older adults being less likely than younger and middle-aged adults to provide high quality decisions, as defined by agreement with expert rankings. However, similar to Queen et al. [14], we hypothesized that decision quality would be predicted by strategic processing measures rather than age or experience. We also expected that search selectivity would emerge as a significant predictor of decision quality, even when age, experience, working memory, and other strategic search measures were included in the model. 

## 2. Methods

### 2.1. Sample

 Data were provided by 176 adults who completed computerized decision making tasks and interviewer-administered surveys. Participants were recruited through published ads and posted fliers and received a cash honorarium for their participation. The adults ranged in age from 18 to 93 years, and about half (42.6%) were men. Reflecting the geographic region from which they were recruited, the majority of the sample (96%) were Caucasian. Most adults (97.6%) had completed high school, with 38.7% having a college degree. Participants completed a 90-minute test battery, including a variety of demographic, social, and cognitive measures; only those of interest to the current report are detailed below. 

### 2.2. Task Materials and Procedure

 All adults completed written informed consent before completing two practice trials. Participants then completed two computerized automobile purchasing decisions for hypothetical others whose needs were presented in short vignettes. The hypothetical vignettes were written such that the needs and resources of the target individuals were implied. Specifically, participants chose an automobile for a middle-aged professional couple who entertained clients regularly and they chose an automobile for a college student who commuted to school and work. The structured design of the scenarios enabled the determination of a correct response and helped to ensure that participants had the same goals for completing the task. The task materials accurately reflected automobiles available on the market, with information provided in a format similar to that found in published guides for consumers. Thus, each of the eight automobiles included information about price, style, appearance, safety, reliability, fuel economy, equipment, and performance. A panel of five experts, using all of the available information, rank ordered the alternatives for appropriateness for each of the vignettes. High interrater reliability was obtained (Kendall's coefficient of concordance, *W* > .80, *P* < .01). In addition, grammatical analyses showed that the reading level of each vignette was appropriate (Fleish index < grade 8.0). Finally, the order of presentation of the two scenarios was counterbalanced across participants to reduce the effects of practice and fatigue. 

### 2.3. Variables and Measures

#### 2.3.1. Individual Characteristics

For the regression analyses, age was used as a continuous variable, although mean age group comparisons were also conducted. Approximately equal numbers of younger adults (*M* = 22.20, SD = 3.44, *n* = 54), middle-aged adults (*M* = 51.45, SD = 11.14, *n* = 60), and older adults (*M* = 76.82, SD = 5.94, *n* = 62) participated. A dichotomous variable (1 = men, 2 = women) was used to index sex.

#### 2.3.2. Cognitive Resources

Two indices of cognitive resources were used. Working memory span was measured using the digits backward test [20], with the standard administration and scoring procedures. The average backward working memory span for the current sample was 4.64 (SD = 1.11, range 2–7). 

 Three dichotomous items assessing experience with the task domain were combined to form a scale. The items included whether a participant had ever made an automobile purchase (70.1%), currently owned an automobile (87.4%), and had ever been asked to give advice to another person considering an automobile purchase (47.9%). The scale ranged from 0 to 3; a mean of 2.06 (SD = .89) was obtained from the current sample.

#### 2.3.3. Similarities across Vignettes

For each decision vignette, participants could view one piece of information at a time during their information search. They were permitted to review as much information as they desired, but were not allowed to take notes. The computer program recorded a variety of measures from which three information search measures were derived: proportion of information searched, order of information searched, and quality of information searched. Paired *t*-tests were used to examine differences across the two vignettes. No significant differences emerged for the amount of information viewed (*t*(175) = 1.33), order of information searched (*t*(175) = 1.13), or selectivity (*t*(175) = 4.81) across the two vignettes. When examining whether participants made one of the two best choices, 48.9% chose correctly in the college student scenario and 45.5% chose correctly in the couple scenario (*t*(175) = .88). Because no mean differences were found between the two vignettes, responses were combined to form a single, averaged index for each of the following measures. 

#### 2.3.4. Task Performance

Amount of information searched was assessed via the proportion of the total number of pieces of information viewed [4, 21]. On average, participants viewed less than half of the available information (*M* = .419, SD = .241). 

 Order of information search (noncompensatory search strategy) was indexed using a ratio of repetition (RR) measure. The RR was originally used in recall tasks to address category clustering, the phenomenon in which people tend to recall a list of randomly-presented words by regrouping the words into categories. Applied to the information matrices, the RR represents the order in which information is viewed. If a decision maker searched within a column of features to compare different alternatives on a single feature, the high RR for features would indicate a noncompensatory search. In the current sample, a mean noncompensatory strategy score of .38 (SD = .28) was obtained. 

 Finally, search selectivity was assessed. Search selectivity concerns which information is viewed, as contrasted with search order. Selectivity was operationalized as the proportion of relevant information the participant searched, with relevance determined by the individual value of a specific piece of information in relation to the needs of the target person. For example, in the college student vignette, the target had little income, no knowledge of autorepair, and needed to commute daily. Thus, the experts indicated that the most relevant features for the student were price, reliability, and fuel economy. For the middle-aged couple who entertained clients and needed an auto for pleasure use, the experts determined that style, safety, and reliability were most relevant. For each vignette, two automobiles were clearly superior, three were of medium quality, and three were inappropriate. Thus, for each vignette, six of the 64 pieces of information were especially relevant. If a participant viewed four of these six pieces of information, the selectivity score would equal .67. In the current sample, a mean selectivity score of .51 (SD = .28) was obtained. 

#### 2.3.5. Decision Quality

Each of the participant's choices were coded to reflect agreement with the rank ordering determined by the expert panels. Points were awarded such that the top choice received a total of 8 points and the least appropriate choice received one point. The points awarded for the two decisions were then combined to form a single index that ranged from 2 to 16 (*M* = 10.95; SD = 3.99).

## 3. Results

### 3.1. Preliminary Analyses

 Means and correlations for the measures are presented in Table 1. To test our hypothesis that age differences would emerge, we conducted a series of one-way analysis of variance tests comparing the three age groups. As shown in Table 2, mean age differences in decision quality were observed. That is, middle-aged adults (*M* = 12.38) offered higher quality decisions than older adults (*M* = 9.50; *F*(2, 173) = 8.68, *P* < .001). 

 Mean age differences also emerged for strategic processing. Specifically, differences were observed for selectivity (*F*(2, 173) = 4.72, *P* = .01) and noncompensatory strategy (*F*(2, 173) = 21.55, *P* < .001). For both selectivity and noncompensatory strategy, younger and middle-aged adults scored higher than older adults. In terms of individual characteristics, differences in experience and working memory were observed. Younger adults had significantly less experience than middle-aged adults (*F*(2, 173) = 4.03, *P* = .019). Middle-aged adults had a higher mean working memory span than older adults (*F*(2, 173) = 3.22, *P* = .042). A nonsignificant age effect was observed for the amount of information viewed (*F*(2, 173) = 2.97, *P* = .054). 

### 3.2. Hypothesis Testing

 To examine our hypothesis regarding the relative contribution of individual characteristics and strategic task performance to the explanation of decision quality, we conducted a 4-step hierarchical linear regression analysis. Model 1 included the demographic indicators of age and gender, entered simultaneously. Model 2 added two individual characteristics, working memory span and domain-specific experience. Model 3 added two measures of strategic task performance, the amount of information viewed, and noncompensatory search strategy. Finally, Model 4 examined the unique contribution of search selectivity to the overall prediction of decision quality. Results for these analyses are presented in Table 3. The equation using only age and gender to explain variance in decision quality reached significance (*F*(2, 173) = 3.12, *P* = .047), but accounted for less than 3% of the variance. Age uniquely accounted for variance in Decision Quality. 

 The equation adding the two everyday problem solving predictors at Step 2 was significant (*F*(4, 171) = 11.72, *P* < .001). Together the four regressors accounted for 19.7% of the variance in Decision Quality. Of the four regressors, only working memory span uniquely contributed to the variance explained. That is, when everyday problem solving predictors were present in the model, age no longer uniquely contributed to the variance in Decision Quality. Adding the task performance measures at Step 3 resulted in an additional 2.0% of explained variance, although the step failed to reach significance (*F*(2, 169) = 2.26, *P* = .11). The model, however, continued to explain significant amounts of variance in decision quality (*F*(6, 169) = 8.68, *P* < .001; *R* ^2^ = .208). Again, only working memory span uniquely contributed to the explained variance.

 Finally, Step 4 examined the contributions of search selectivity. Both the step (*F*(1, 168) = 46.76, *P* < .001) and the model (*F*(7, 168) = 16.14, *P* < .001) reached significance. Search selectivity accounted for an additional 16.6% of the variance beyond that explained by age, gender, working memory, experience, amount of information viewed, and noncompensatory search strategy. The final model accounted for 37.7% of the variance in Decision Quality. In the final model, larger working memory span, more information viewed, and higher search selectivity uniquely contributed to the variance accounted for in Decision Quality. 

 A series of exploratory analyses was conducted to examine potential interactions with age and with working memory among the predictor set. Predictors were centered and entered into a first step of a hierarchical regression. The interaction terms were then entered in a second step. In no case did an interaction term add to the variance accounted for in Decision Quality. 

## 4. Discussion

 Our process-tracing technique and the known quality of information allows an examination of the adults' performance at multiple stages of decision making, including the selection of information and arriving at a solution. Our results show that the quality of the information one chooses directly affects the quality of the decisions one makes. While this finding is certainly intuitive, we empirically demonstrated this association. This is an important contribution because the finding can be used to guide training and intervention programs to increase the decision making competence of adults. Specifically, by being able to determine the relative value of a single piece of information, we can assess how selective a problem solver is in her search processes. Moreover, by knowing the value of each piece of information, we can determine decision quality. 

 In addition to the important role of search selectivity, our results show an important role for working memory span and search thoroughness. As an initial investigation into decision quality, we chose a consumer task in which identifying the relevance of the information and decision quality was somewhat straight-forward. Given previous findings in these domains [4, 14], it is surprising that mean age differences emerged. Interestingly, age contributed little to the explanation of decision quality. Together, these results suggest that we can begin to explore ways in which to improve decision making quality. Implications for each of these avenues, as well as how our results relate to current theoretical frameworks are discussed in the following sections. 

### 4.1. Individual Characteristics

 Several mean age differences were observed in the current study. Specifically, older adults had smaller working memory spans, searched less selectively, were less likely to use a noncompensatory strategy, and were less likely to choose one of the top two alternatives. Despite these mean differences, however, our regression results find no significant contribution of chronological age when additional measures are included in the equation. This result reaffirms the idea that chronological age is a poor proxy for cognitive ability. 

 Research demonstrates that decision making is affected by a variety of cognitive processes such as working memory and speed, which can be improved with training [13]. Our study adds to this literature by demonstrating that working memory is not only related to information search but also exerts direct effects on decision quality. Thus, it may be possible to improve decision making competence by intervening at the level of these cognitive processes. 

 The literature also suggests an important role for experience [9, 15, 17]. Among our community-dwelling nonexperts, experience did not contribute to decision quality. We suspect that our measure of experience was too crude to adequately assess the effects of experience on decision quality. Our index included three dichotomous items for which a large number of participants answered affirmatively. Thus, the failure of experience to relate to decision quality could be a function of ceiling effects on the measure. A second explanation for the failure of experience to explain variance in decision quality may relate to the nature of that experience. As Patrick and Strough [9] note, recency of experience may be particularly important. We encourage additional research that examines the role of prior experience in problem solving quality, particularly whether experience operates similarly across adulthood. 

### 4.2. Task Performance

 Decision making research often focuses on the thoroughness of information search. Typical results show that older adults use less information than younger or middle-aged adults [4]. In our analyses, no significant age differences in thoroughness emerged. Moreover, in the regressions, thoroughness only uniquely contributed to the variance in decision quality when selectivity was present in the equation. Although additional investigation is necessary, this relation may be due to potentially dual routes to acquiring the relevant information. People who are highly selective may view little of the total information, thus scoring lower on measures of thoroughness. However, through increased thoroughness, people increase the likelihood that they will view the relevant information. Thus, highly selective searchers may view less total information as they focus on the most relevant information. In contrast, highly thorough searchers are also likely to acquire the most important information. Overall, however, our results show that quantity is less important than the quality of the information. Additional investigation regarding the association between amount of information searched and the quality of that information is advised. 

 Both the age differences in search strategy and the relative importance of the use of a noncompensatory strategy in the regression analyses are intriguing. Together, these two sets of analyses suggest that age differences in problem solving efficiency may be a direct function of problem solving strategy. Such a conclusion is consistent with a range of everyday problem solving studies showing strong age differences in problem solving strategy [2]. The current data are able to link these strategic differences to differences in the quality of responses. Thus, the analyses demonstrate that age differences in strategic processing are important because they directly relate to age differences in solution quality. 

The measure of strategic processing, drawn from the decision making literature, was a global summary of how much of an individual's search could be characterized as noncompensatory. Noncompensatory strategies are generally viewed as an adaptive response to working memory overload [4, 18]. It is interesting that older adults demonstrated smaller working memory spans but did not show a high use of noncompensatory search. Again, these results suggest two avenues for intervention: at the level of working memory capacity and at the level of task performance. 

 Finally, although the task offers several advantages to studying both problem solving process and outcome, it does have limitations. Specifically, participants could only view one piece of information at a time during their information search. They were permitted to review as much information as they desired, but were not allowed to take notes. In the real-world, consumers may adopt more external aids to facilitate their information search. However, the process tracing technique present in the current task offers sufficient advantages to offset this limitation. That is, the technique allows an examination of which information is viewed and in what order. These two aspects of information search are the essence of strategic processing. 

### 4.3. Theoretical Implications and Future Directions

 In our consumer problem, age contributed little to decision quality. This finding is consistent with previous research [4, 14] in this domain. A major contribution of our work rests with the computation of Decision Quality. Both Johnson [4] and Queen et al. [14] relied on the individuals' preferences as an outcome measure. Our conceptualization of decision quality focuses on making a decision for others with stated and implied needs. This difference is important in that it allows us to determine information quality and thus, search selectivity. 

 We strongly advocate for an extension of both the process-tracing approach and the information quality aspect to a wider range of domains. Significant newer research is examining the tradeoff between affective and logical goals [10], but because these domains may exhibit marked idiosyncrasies, it might be especially challenging to examine information quality in vignettes featuring an interpersonal problem [22]. Although work and wisdom [23] might inform such efforts. Such interpersonal expertise may be similar to the processes used by experts in instrumental domains and warrants additional research attention. Vignettes could be designed to include both emotion-laden and instrumental information. Investigators could then directly examine the type of information that adults use when making decisions in a variety of domains, including interpersonal, health [15], and financial [13]. 

In summary, it is important to develop tasks and measures that reflect the everyday competence of older adults [13] so that we can identify individuals in need of decision making support. Rather than cataloging age differences; however, it is necessary to link age differences in process to some difference in performance outcome. We present a task and measures that may significantly advance the field's progress toward that goal. By focusing on the quality of an adult's decision in a hypothetical vignette, differences in strategy become more meaningful. We encourage additional and independent investigation using this class of tasks, and we look forward to refinements of the measures of strategy, selectivity, and decision quality.

## Figures and Tables

**Table 1:** Descriptive statistics and correlations.

		Mean	SD	1	2	3	4	5	6	7	8
(1)	Age	51.41	23.45	1.0	.056	−.132	.164*	−.177*	−.396**	−.183*	−.175*
(2)	Gender (1 = men, 2 = women)	1.57	0.50	—	1.0	−.012	−.197**	.059	−.099	.050	−.073
(3)	Backward digit span	4.64	1.11	—	—	1.0	.022	.116	.016	.293**	.443**
(4)	Experience	2.06	0.89	—	—	—	1.0	−.108	.028	−.083	.036
(5)	Proportion of information viewed	0.42	0.24	—	—	—	—	1.0	−.194**	.618**	.196**
(6)	Noncompensatory search	0.38	0.28	—	—	—	—	—	1.0	−.088	−.013
(7)	Search selectivity	0.51	0.28	—	—	—	—	—	—	1.0	.525**
(8)	Decision quality	10.95	3.99	—	—	—	—	—	—	—	1.0

*P < .05, **P < .01.

**Table 2:** Mean age group differences.

	Group A	Group B	Group C		
	Younger (*n* = 54)	Middle aged (*n* = 60)	Older (*n* = 62)	*F*	Post Hoc
Age	22.20	51.45	76.82	737.22***	A < B < C
Experience	1.78	2.22	2.15	4.03*	A < B
Working memory	4.65	4.88	4.38	3.23*	B > C
Proportion viewed	0.47	0.42	0.37	2.97*	
Noncompensatory search	0.49	0.45	0.21	21.55***	A and B > C
Search selectivity	0.55	0.56	0.42	4.72**	A and B > C
Decision quality	11.03	12.38	9.50	8.68***	B > C

*P ≤ .05, **P ≤ .01, ***P ≤ .001.

**Table 3:** Linear regression predicting decision quality (*n* = 176).

	b	SE_b	B
Step 1			
Age	−.029	.013	−.172*
Gender	−.513	.602	−.064

Step 2			
Age	−.021	.012	−.122
Gender	−.437	.558	−.054
Experience	161	.315	.036
Working memory	1.532	.246	.425***

Step 3			
Age	−.021	.013	−.124
Gender	−.517	.556	−.064
Experience	.222	.314	.050
Working memory	1.480	.246	.411***
Proportion of information viewed	2.077	1.192	.125
Noncompensatory search	−.752	1.108	−.052

Step 4			
Age	−.017	.012	−.099
Gender	−.597	.493	−.074
Experience	.256	.279	.057
Working memory	1.051	.227	.292***
Proportion of information viewed	−3.164	1.306	−.191*
Noncompensatory search	−.792	.983	−.055
Search selectivity	7.676	1.123	.543***

Step  1: *F*(2,173) = 3.12*; *R* ^2^ = .027. Step  2: *F*(2,171) = 19.65***; model *F*(4,171) = 11.72***; *R* ^2^ = .197. Step  3: *F*(2,169) = 2.26; model *F*(6,169) = 8.68***; *R* ^2^ = .208. Step  4: *F*(1,168) = 46.76***; model *F*(7,168) = 16.14***; *R* ^2^ = .377.
